# TIGAR/AP-1 axis accelerates the division of *Lgr5*^−^ reserve intestinal stem cells to reestablish intestinal architecture after lethal radiation

**DOI:** 10.1038/s41419-020-2715-6

**Published:** 2020-07-06

**Authors:** Fei Chen, Yushuo Zhang, Songling Hu, Xiaolin Shi, Zhongmin Wang, Zicheng Deng, Longxin Lin, Jianghong Zhang, Yan Pan, Yang Bai, Fenju Liu, Haowen Zhang, Chunlin Shao

**Affiliations:** 1 Institute of Radiation Medicine, Fudan University, Shanghai, 200032 China; 2 State Key Laboratory of Radiation Medicine and Protection, School of Radiation Medicine and Protection, Medical College of Soochow University, Collaborative Innovation Center of Radiological Medicine of Jiangsu Higher Education Institutions, Jiangsu Provincial Key Laboratory of Radiation Medicine and Protection, Suzhou, 215123 China; 3 Department of Interventional Radiology, Ruijin Hospital, Shanghai Jiao Tong University School of Medicine, Shanghai, 200025 China; 4 Department of Interventional Radiology, The Third Affiliated Hospital of the Medical College of Shihezi University, Xinjiang, 832008 China

**Keywords:** Intestinal stem cells, Stem-cell research

## Abstract

During radiologic or nuclear accidents, high-dose ionizing radiation (IR) can cause gastrointestinal syndrome (GIS), a deadly disorder that urgently needs effective therapy. Unfortunately, current treatments based on natural products and antioxidants have shown very limited effects in alleviating deadly GIS. Reserve intestinal stem cells (ISCs) and secretory progenitor cells are both reported to replenish damaged cells and contribute to crypt regeneration. However, the suppressed β-catenin/c-MYC axis within these slow-cycling cells leads to limited regenerative response to restore intestinal integrity during fatal accidental injury. Current study demonstrates that post-IR overexpression of TIGAR, a critical downstream target of c-MYC in mouse intestine, mounts a hyperplastic response in *Bmi1-creERT*^+^ reserve ISCs, and thus rescues mice from lethal IR exposure. Critically, by eliminating damaging reactive oxygen species (ROS) yet retaining the proliferative ROS signals, TIGAR-overexpression enhances the activity of activator protein 1, which is indispensable for initiating reserve-ISC division after lethal radiation. In addition, it is identified that TIGAR-induction exclusively gears the *Lgr5*^−^ subpopulation of reserve ISCs to regenerate crypts, and intestinal TIGAR-overexpression displays equivalent intestinal reconstruction to reserve-ISC-restricted TIGAR-induction. Our findings imply that precise administrations toward *Lgr5*^−^ reserve ISCs are promising strategies for unpredictable lethal injury, and TIGAR can be employed as a therapeutic target for unexpected radiation-induced GIS.

## Introduction

Unexpected radiation exposure during terrorist events (e.g., the use of “dirty bombs”), industrial or nuclear accidents (such as the nuclear disasters in Chernobyl and Fukushima) is a current and continuing threat to the future. Under homeostatic conditions, the rapid turnover of the intestinal epithelium is driven by leucine-rich repeat-containing G protein-coupled receptor 5 (*Lgr5*)^high^ intestinal stem cells (ISCs), which are especially vulnerable to high-dose ionizing radiation (IR)^1,2^. A dose of 15 gray (Gy) of radiation is sufficient to abrogate the proliferative output of these mitotically active *Lgr5*^high^ ISCs, and thus causes severe acute damage of the epithelial integrity^2^. Within 7 days of high-dose IR exposure, mice suffered from diarrhea, malabsorption and weight loss always die with complications known as gastrointestinal syndrome (GIS). Although prophylactic administrations have demonstrated some desirable effects on preventing stem cell exhaustion and epithelial disintegration induced by high-dose IR exposure^3–5^, the current post-IR treatments based on natural products and antioxidants have shown very limited effects on reversing stem cell death and the deadly GIS^6–8^.

Besides the high-proliferating and radiosensitive *Lgr5*^high^ ISCs (i.e., crypt base columnar cells (CBCs)), a slow-cycling and injury-resistant pool of stem cells could be arisen to divide when the CBCs are depleted^9,10^. These rare “+4” position cells mainly include the reserve ISCs marked by lineage tracing analysis with *polycomb complex protein 1* (*Bmi1*)*-creERT*^11,12^ and *Lgr5*^+^ label-retaining secretory progenitor cells which are regarded functionally distinct from reserve ISCs^13,14^. These radioresistant “+4” position cells are low-proliferative under homeostasis, while become proliferative from 3–4 days after high-dose radiation^15,16^. However, during lethal IR exposure, the CBCs are exhausted rapidly and the intestinal epithelium always disintegrates around 5 days after radiation, which happens even prior to effective “+4”-position-cell division and crypt regeneration. Hence, further elucidation of the mechanisms leading these quiescent cells to division after lethal IR-injury is required for mitigating fatal GIS.

The Wnt/β-catenin/c-MYC axis plays a central role in regulating the division of ISCs. However, the suppressed β-catenin/c-MYC pathway within “+4” position cells results in limited regenerative response within 3 days after lethal radiation^16^. Therefore, targeting β-catenin/c-MYC signal after lethal IR-injury may be potential countermeasures for accelerating the regeneration of these quiescent cells and the intestinal epithelium. TP53-induced glycolysis and apoptosis regulator (TIGAR), a downstream target of c-MYC in mouse intestinal crypts^17^, has been indicated to be a critical scavenger of reactive oxygen species (ROS), which promotes DNA damage repair and cellular redox balance during genotoxic stress^18,19^.

In the present study, we demonstrate that overexpression of TIGAR may be promising in ameliorating the intestinal architecture and survival during unpredictable lethal injury. Mechanistically, TIGAR acts as a turn-on switch that facilitates cell division of *Lgr5*^−^ reserve ISCs in an activator protein 1 (AP-1) dependent manner, which remedies the β-catenin/c-MYC-inhibited “defect” of these cells and gears crypt regeneration efficiently after lethal IR-injury.

## Results

### Restricted TIGAR-overexpression in reserve ISCs mitigates lethal GIS

**Fig. 1:**
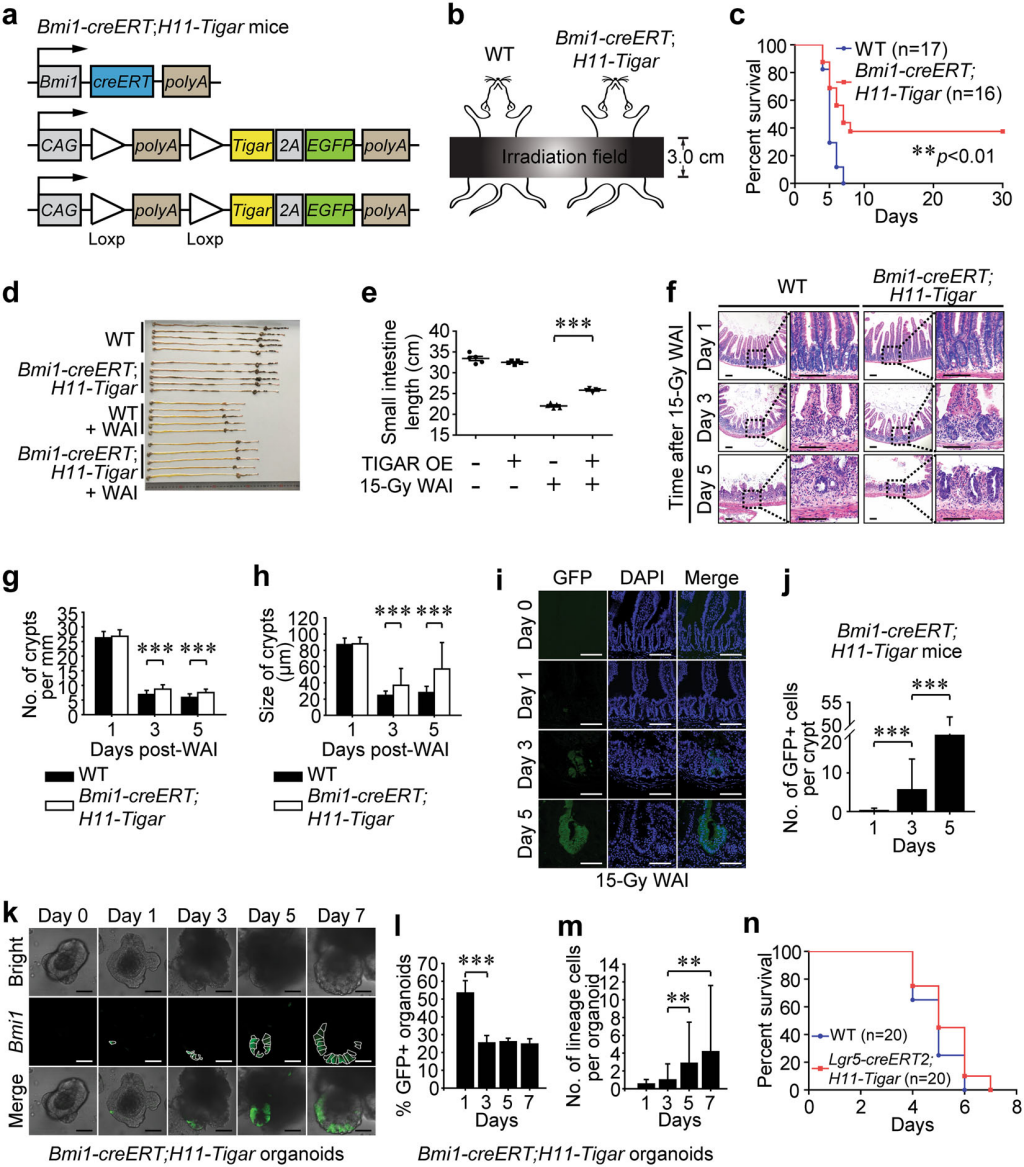
Restricted TIGAR-overexpression in reserve ISCs mitigates radiation-induced lethal GIS. **a** Gene-targeting strategy for *Bmi1-creERT;H11-Tigar* mice. **b** Schematic diagram of the WAI exposure field for *Bmi1-creERT;H11-Tigar* and WT mice. *H11-Tigar* mice serve as WT control. **c** Kaplan–Meier survival analysis of WT and *Bmi1-creERT;H11-Tigar* mice after 15-Gy WAI. **d** Image of small intestines 5 days post 15-Gy WAI. **e** Length of small intestines from TIGAR-overexpressing (OE) *Bmi1-creERT;H11-Tigar* mice and their WT cohorts 5 days after 15-Gy WAI. **f** Representative images of H&E staining of small intestines after 15-Gy WAI. Six sections per mouse, *n* = 3 animals. Scale bars = 100 μm. **g** Number of crypts per millimeter after 15-Gy WAI. Values are expressed as mean ± SD. **h** Size of crypts at indicated time post-WAI. Values are expressed as mean ± SD. **i** Representative frozen-sections of proximal intestine derived from *Bmi1-creERT;H11-Tigar* mice. Fluorescence microscopy illustrates the GFP-positive crypts at indicated time post-IR. Six sections per mouse, *n* = 3 animals. Scale bars = 100 μm. **j** Number of GFP-positive cells per crypt from *Bmi1-creERT;H11-Tigar* mice. Values are expressed as mean ± SD. **k** Lineage tracing analysis of organoids derived from *Bmi1-creERT;H11-Tigar* mice. Fluorescence microscopy illustrates the *Bmi1*^*+*^ lineage (GFP) at indicated time post-IR. White dashed lines illustrate a single cell. Scale bars = 50 μm. **l** Percentage of GFP-positive organoids from *Bmi1-creERT;H11-Tigar* mice. **m** Number of GFP-positive cells per organoid. Values are expressed as mean ± SD. **n** Kaplan-Meier survival analysis of *Lgr5-creERT2;H11-Tigar* mice after 15-Gy WAI. **p < 0.01, ***p < 0.001.

TIGAR has been indicated to be crucial for efficient CBC proliferation under genotoxic stress^20^. However, the actively cycling CBCs are too radiosensitive to be rescued by post-IR treatment, always undergoing apoptosis within 12 h after lethal IR exposure^20^. Given that both of the reserve ISCs and *Lgr5*^+^ label-retaining secretory progenitor cells contribute to intestinal regeneration, mice containing the *loxp-stop-loxp-Tigar* cassette allele were crossed with *Bmi1-creERT* mice and *Lgr5-EGFP-IRES-creERT2* (*Lgr5-creERT2*) mice, respectively (Fig. 1a; and Supplementary Fig. 1a). Immediately after 15-Gy whole abdominal irradiation (WAI) (Fig. 1b), restricted TIGAR-induction within *Bmi1-creERT*^+^ or *Lgr5-creERT2*^*+*^ cells was performed upon a single intraperitoneal injection of tamoxifen. Critically, *Bmi1-creERT;H11-Tigar-2A-EGFP* (*Bmi1-creERT;H11-Tigar*) mice revealed a significantly increased survival of 37.5% beyond 30 days post-WAI in comparison with the wild-type (WT) cohorts with 100% mortality within 7 days post-IR (Fig. 1c). Consistently, inducible TIGAR-overexpression dramatically attenuated the intestine shortening 5 days post-WAI (Fig. 1d, e). Histopathological analysis of the proximal small intestines (Fig. 1f) further confirmed that specific overexpression of TIGAR in reserve ISCs largely reversed crypt loss (Fig. 1g) and reduction of crypt size (Fig. 1h) after lethal irradiation. Lineage tracing analysis indicated a growing *Bmi1-creERT*^+^ lineage in regenerative crypts after lethal IR-injury (Fig. 1i, j). Using cultured mouse miniguts in vitro, i.e., epithelial organoids that preserved the in vivo cell-type distribution and kinetics of intestinal crypts and villi^21,22^, it was found that around 24.6 ± 3.05% of organoids derived from *Bmi1-creERT;H11-Tigar* mice were robustly labeled with GFP at 7 days after 12-Gy irradiation (a lethal dose for cultured intestinal organoids in vitro^23^) (Fig. 1k, l). The number of GFP-positive cells per organoid was 1.00 ± 1.80, 2.86 ± 4.61 and 4.16 ± 7.44 in *Bmi1-creERT;H11-Tigar* cohort by day 3, day 5 and day 7 post-IR, respectively (Fig. 1m), suggesting a continuous expansion of *Bmi1-creERT*^+^ population. By comparison, the restricted TIGAR-induction in *Lgr5-creERT2*^+^ cells in vivo revealed no discernible quantitative effects on reversing intestinal disintegration (Supplementary Fig. 1b–e) and WAI-induced lethality (Fig. 1n) when compared with their WT cohorts. In vitro lineage tracing analysis also demonstrated that the restricted TIGAR-induction within *Lgr5-creERT2*^+^ cells failed to promote intestinal organoid regeneration (Supplementary Fig. 1f, g), and *Lgr5*^+^ cells along with their progeny cells in *Lgr5-creERT2;H11-Tigar-2A-EGFP* (*Lgr5-creERT2;H11-Tigar*) organoids were abolished from 2 days onward after 12-Gy irradiation (Supplementary Fig. 1h). Altogether, our results disclosed that the restricted TIGAR-induction within *Bmi1-creERT*^+^ reserve ISCs but not *Lgr5*^+^ label-retaining secretory progenitors could gear intestinal regeneration and ameliorate mouse survival after lethal WAI.

### TIGAR-induction exclusively gears *Lgr5*^*-*^ reserve ISCs to regenerate crypts

**Fig. 2:**
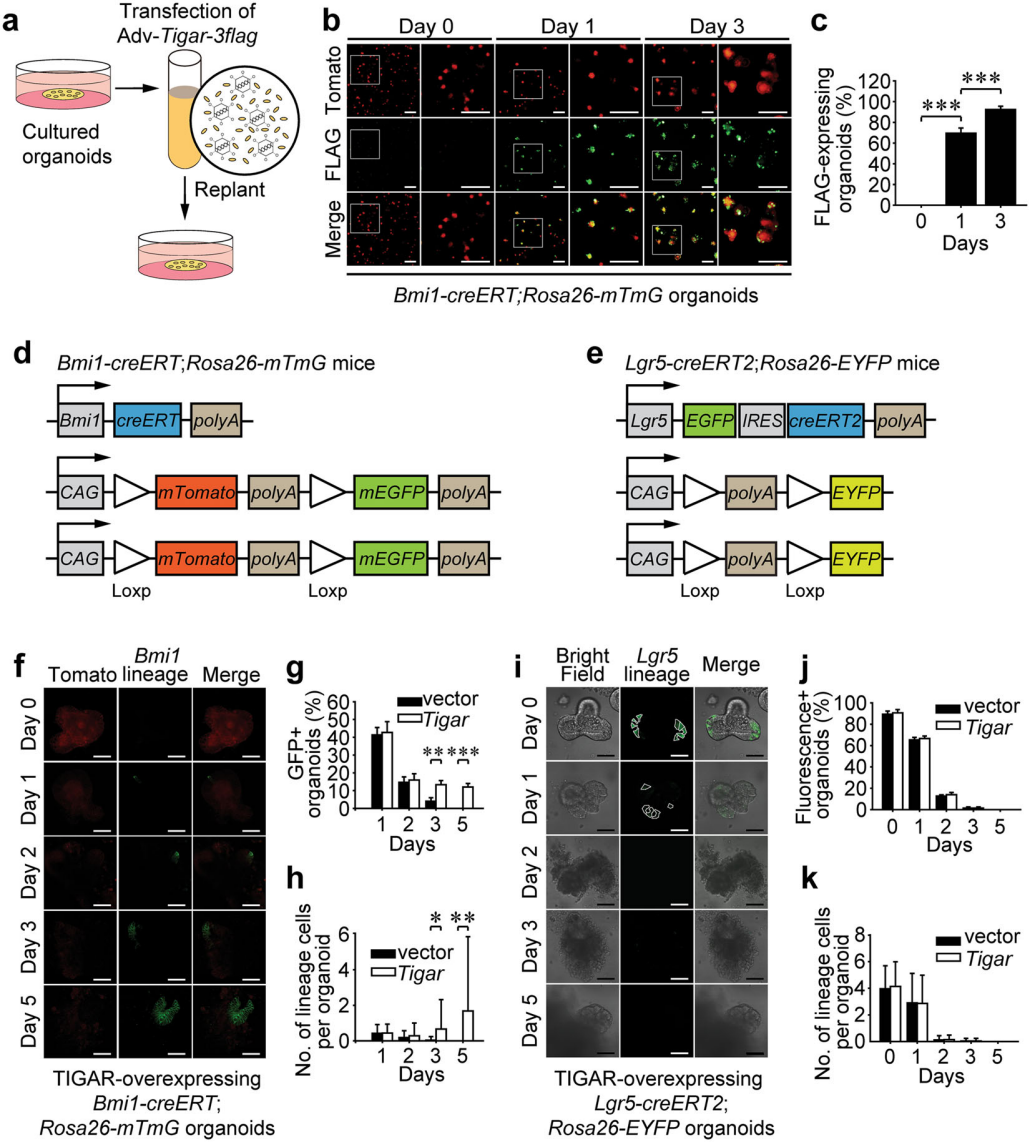
TIGAR-induction exclusively gears *Lgr5*^−^ reserve ISCs to regenerate crypts. **a** Schematic diagram depicting the transfection process of TIGAR-overexpressing adenovirus, Adv-*CMV*-*Tigar*-*3flag*. **b** Immunofluorescence assay illustrates the FLAG-expressing organoids at the indicated time after transfection. **c** Percentage of FLAG-expressing organoids at indicated time after transfection. **d**, **e** Gene targeting strategies for *Bmi1-creERT;Rosa26-mTmG* mice and *Lgr5-creERT2;Rosa26-EYFP* mice. **f, i** Lineage cell tracing with organoids derived from *Bmi1-creERT;Rosa26-mTmG* mice and *Lgr5-creERT2;Rosa26-EYFP* mice. Scale bars = 50 μm. **g**, **j** Percentage of fluorescence-positive organoids from *Bmi1-creERT;Rosa26-mTmG* mice and *Lgr5-creERT2;Rosa26-EYFP* mice at indicated time after 12-Gy irradiation. **h**, **k** Number of fluorescence-positive cells per organoid from *Bmi1-creERT;Rosa26-mTmG* mice and *Lgr5-creERT2;Rosa26-EYFP* mice at indicated time after 12-Gy irradiation. Values are expressed as mean ± SD. *p < 0.05, **p < 0.01, ***p < 0.001.

By asymmetric division, a single reserve ISC could generate a daughter cell and an *Lgr5*^+^ CBC to replenish the active stem cell compartment^15^. The active CBCs then either generate transit-amplifying (TA) cells, which divide rapidly to produce large quantities of enterocytes, or differentiate into secretory progenitor cells which commit to Paneth cells, goblet cells, or enteroendocrine cells^10^. Principal component analysis of single ISCs reveals clear differences of reserve ISCs in comparison to *Lgr5*^high^ CBCs^24^. However, nearly 20% of the *Bmi1-creERT*^+^ population is reported to exhibit an “*Lgr5*^+^” identity, a transition state between the quiescent reserve ISCs and the active CBCs^10,24^. In order to identify whether TIGAR-induced crypt regeneration originated in the *Lgr5*^+^ subpopulation of reserve ISCs or the *Lgr5*^-^ ones, we sought to perform in vitro TIGAR-overexpression by adenovirus transfection. The transfection was performed by mechanical separating the organoids from Matrigel (Fig. 2a), and TIGAR was able to be efficiently overexpressed in 69.4 ± 5.08% of organoids at 1 day post transfection (Fig. 2b, c). During lineage cell tracing analysis, organoids derived from either *Bmi1-creERT;Rosa26-mTmG* mice (Fig. 2d) or *Lgr5-creERT2;Rosa26-EYFP* mice (Fig. 2e) were exposed to 12-Gy IR in vitro and then transfected immediately with an adenoviral vector expressing TIGAR. The 4-hydroxytamoxifen (4-OHT) stimulation was performed soon after replanting the organoids, and the progeny of total *Bmi1-creERT*^*+*^ reserve ISCs or offspring of *Lgr5-creERT2*^*+*^ subpopulations could be marked by fluorescence. As expected, TIGAR-overexpression facilitated the *Bmi1-creERT*^+^ cell-derived lineage following irradiation (Fig. 2f–h). Nonetheless, the *Lgr5-creERT2*^+^ subpopulation was not benefited by TIGAR-overexpression since the fluorescence was undetectable in the live organoids 3–5 days post IR (Fig. 2i–k). Consequently, it could be speculated that TIGAR-induction within *Bmi1-creERT*^+^ ISCs predominantly promoted the *Lgr5*^-^ reserve ISCs, rather than the *Lgr5*^+^ subpopulations, to regenerate thus ameliorated intestinal integrity after lethal IR exposure.

### TIGAR-induced crypt regeneration is AP-1 dependent

**Fig. 3:**
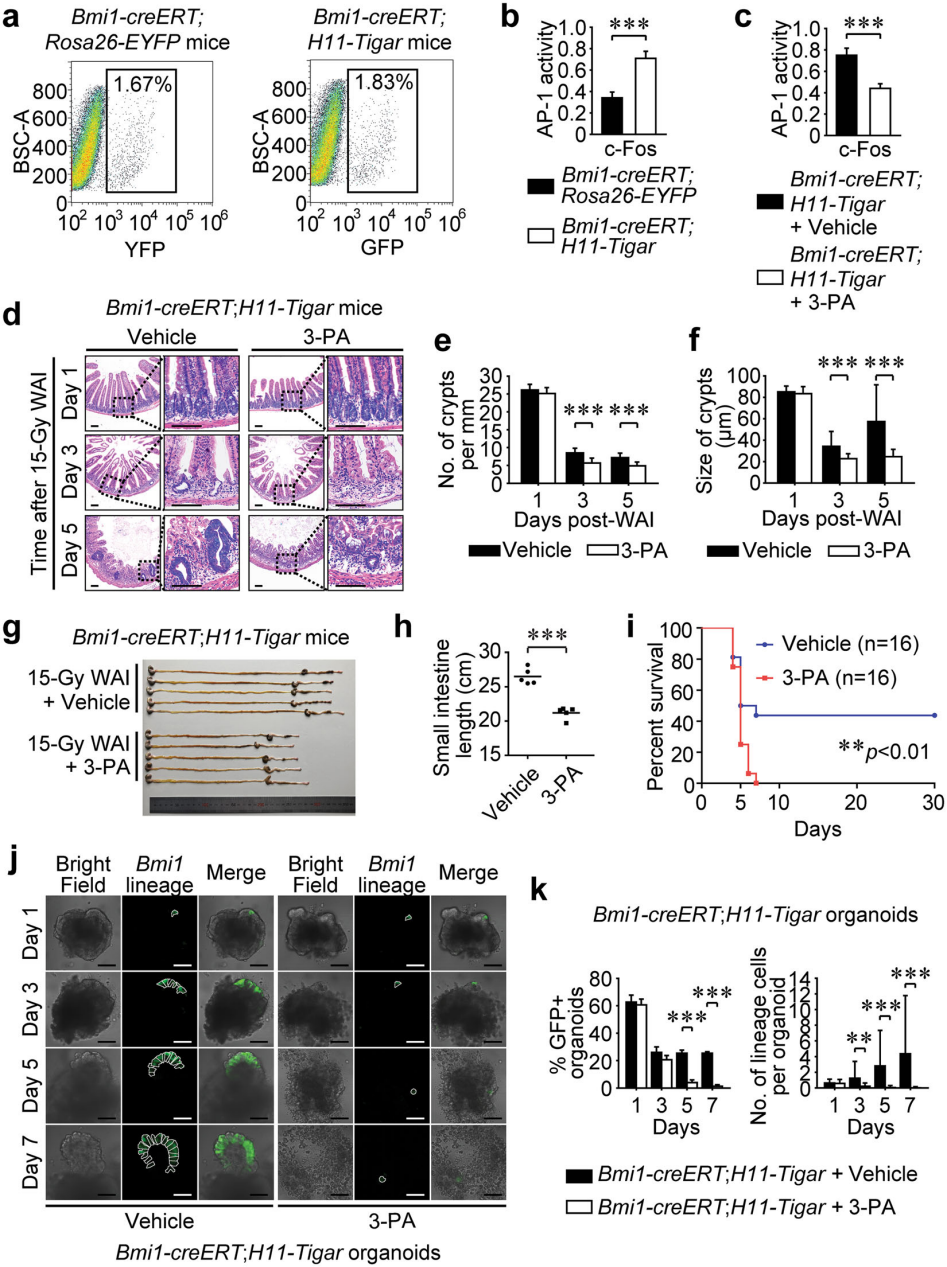
TIGAR-induced crypt regeneration is AP-1 dependent. **a** Representative FACS plots of *Bmi1-creERT*^*+*^ cells isolated from intestinal crypts of *Bmi1-creERT;Rosa26-EYFP* mice (left) or *Bmi1-creERT;H11-Tigar* mice (right) 24 h after 15-Gy WAI and the subsequent tamoxifen injection. **b** The c-Fos/AP-1 activity within isolated *Bmi1-creERT*^*+*^ cells 24 h after WAI. Cells are derived from *Bmi1-creERT;H11-Tigar* mice or *Bmi1-creERT;Rosa26-EYFP* mice which serve as a control. Values are expressed as mean ± SD. **c** *Bmi1-creERT*^*+*^ cells are isolated from intestinal crypts by FACS one day post-WAI, and the c-Fos/AP-1 activity within isolated *Bmi1-creERT*^*+*^ cells is determined. Values are expressed as mean ± SD. **d** Representative images of H&E staining of small intestines from *Bmi1-creERT;H11-Tigar* mice (left panel) and 3-PA treated ones (right panel) after 15-Gy WAI. Six sections per mouse, *n* = 3 animals. **e** Number of crypts per millimeter. Values are expressed as mean ± SD. **f** Size of crypts after 15-Gy IR. Values are expressed as mean ± SD. **g** Image of small intestines 5 days post-IR. **h** Length of small intestines. **i** Kaplan–Meier survival analysis of *Bmi1-creERT;H11-Tigar* mice (Vehicle) and 3-PA treated cohorts (3-PA) after 15-Gy WAI. **j** Lineage cell tracing analysis of organoids derived from *Bmi1-creERT;H11-Tigar* mice. White dashed lines indicate a single cell. Scale bars = 50 μm. **k** Percentage of fluorescence-positive organoids and the number of GFP-positive cells per organoid from *Bmi1-creERT;H11-Tigar* mice. Values are expressed as mean ± SD. **p < 0.01, ***p < 0.001.

Our previous study has reported that TIGAR has a function of providing antioxidant defense and maintaining cellular redox balance, which potentially contributes to activating the nuclear transcriptional factors such as AP-1^19^. In the present study, we undertook fluorescence-activated cell sorting (FACS) to enrich *Bmi1-creERT*^+^ reserve ISCs 1 day after 15-Gy WAI (Fig. 3a), so that the activity of AP-1 in the collected *Bmi1-creERT*^+^ cells could be examined. As shown in Fig. 3b, the c-Fos/AP-1 activity was significantly increased in TIGAR-overexpressing reserve ISCs, suggesting that AP-1 might be pivotal in TIGAR-induced intestinal hyperplastic response after lethal radiation. For further confirmation, a specific inhibitor of AP-1, 3-(5-(4-(cyclopen-tyloxy)-2-hydroxybenzoyl)-2-((3-hydroxybenzo[d]isoxazol-6-yl) methoxy) phenyl) propanoic acid (3-PA)^25,26^ was used in vivo (Supplementary Fig. 2a). It was found that 3-PA potently inhibited the AP-1 activity in TIGAR-overexpressing *Bmi1-creERT*^+^ reserve ISCs after 15-Gy WAI (Fig. 3c). Consistently, AP-1 abolishment dramatically abrogated TIGAR-induced intestinal regeneration (Fig. 3d–h) and attenuated mouse survival after lethal irradiation (Fig. 3i). Notably, the *Bmi1-creERT*^+^ lineage cells per TIGAR-overexpressing organoid as well as the percentage of GFP-positive organoids were significantly diminished by 3-PA treatment in vitro (Fig. 3j, k; and Supplementary Fig. 2b), suggesting that the AP-1 activity was indispensable for initiating TIGAR-geared division of reserve ISCs after deadly IR-injury.

### TIGAR accelerates reserve ISCs toward division by limiting damaging ROS

**Fig. 4:**
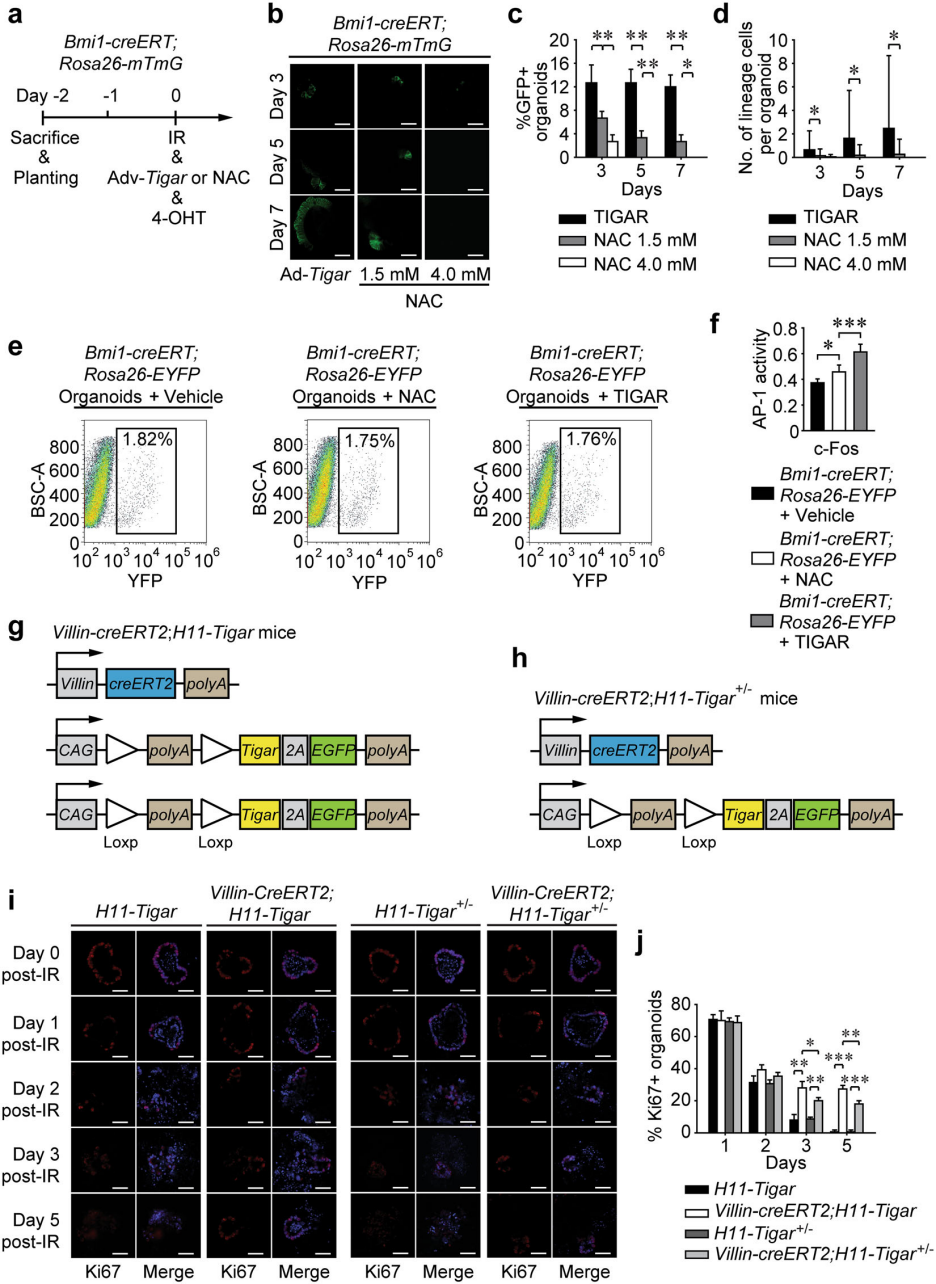
TIGAR accelerates reserve ISCs toward division by limiting damaging ROS. **a** Schematic diagrams show the experimental strategy for co-treatment with irradiation and *Tigar*-overexpressing adenovirus as well as irradiation and NAC (1.5 mM or 4.0 mM) in vitro. **b** Lineage cell tracing of organoids derived from *Bmi1-creERT;Rosa26-mTmG* mice. Fluorescence microscopy shows the GFP-positive organoids at indicated time post-IR. Scale bars = 50 µm. **c** Number of GFP-positive organoids at indicated time post-IR. Values are expressed as mean ± SD. **d** Number of GFP-positive cells per organoid from *Bmi1-creERT;Rosa26-mTmG* mice. Values are expressed as mean ± SD. **e** Representative FACS plots of *Bmi1-creERT*^*+*^ cells isolated from intestinal organoids 24 h after irradiation. **f** The c-Fos/AP-1 activity within isolated *Bmi1-creERT*^*+*^ cells. Values are expressed as mean ± SD. **g** Gene targeting strategy for homozygous *Villin-creERT2;H11-Tigar*^+/+^ (*Villin-creERT2;H11-Tigar*) mice. **h** Gene targeting strategy for heterozygous *Villin-creERT2;H11-Tigar*^+/-^ mice. **i** Immunofluorescence assay for organoids derived from *Villin-CreERT2;H11-Tigar* and *Villin-creERT2;H11-Tigar*^+/-^ mice as well as their WT cohorts. Ki67 is colored in red and DAPI in blue. Scale bars = 50 μm. **j** Percentage of Ki67 positive organoids. Values are expressed as mean ± SD. *p < 0.05, **p < 0. 01, ***p < 0.001.

It was noteworthy that redundant TIGAR activity failed to gear reserve-ISC division during homeostatic conditions. Upon a pulse of 4-OHT in vitro and TIGAR-overexpression within reserve ISCs, the morphology and dynamics of TIGAR-overexpressing organoids remained the same as that of WT cohorts (Supplementary Fig. 3a, b). The reason might be attributed to another “initiating signal”, which was essential for gearing reserve ISCs toward regeneration. It was reported that the proliferative ROS signal was pivotal for CBC division and proliferation^27^. With the administration of N-acetyl L-cysteine (NAC), a traditional antioxidant which indiscriminately scavenged the damaging ROS and pro-proliferating ROS, the regeneration of reserve ISCs was examined to determine whether IR-induced pro-proliferation ROS acted as “initiating signal” in accelerating reserve-ISC division. As shown in Fig. 4a–d, NAC treatment could only drive the *Bmi1-creERT*^+^ cell division to a limited extent, which was far away from that of TIGAR-overexpression did. In vitro analysis also revealed that the AP-1 activity within *Bmi1-creERT*^+^ cells after 12-Gy irradiation was modestly enhanced by the NAC administration, with the degree lagging far behind the TIGAR-overexpressing cohort’s (Fig. 4e, f). To confirm whether TIGAR-induced crypt regeneration was in a dose-dependent pattern, intestinal organoids derived from both homozygous *Villin-creERT2;H11-Tigar*^+/+^ (*Villin-creERT2;H11-Tigar*) mice (Fig. 4g) and heterozygous *Villin-creERT2;H11-Tigar*^+/−^ mice (Fig. 4h) were irradiated and stimulated by 4-OHT immediately post-IR. Ki67-based immunofluorescence assay illustrated that both homozygous and heterozygous TIGAR-overexpressing miniguts moved to proliferative phases that were notable at 3–5 days after irradiation (Fig. 4i, j). The degree, however, in the homozygous TIGAR-overexpressing organoids was considerably higher than that in heterozygous ones (Fig. 4i, j), whose TIGAR expression level was between WT organoids and homozygous organoids. These data further confirmed that IR-induced pro-proliferating ROS, which was not scavenged by TIGAR, might be a critical “initiating signal” for gearing reserve ISCs toward regeneration. This mechanism also explained why preclinical treatments simply based on traditional antioxidants had very limited effects on reversing intestinal disintegration and lethal GIS.

### Intestinal TIGAR-overexpression displays equivalent reconstruction to restricted TIGAR-induction

**Fig. 5:**
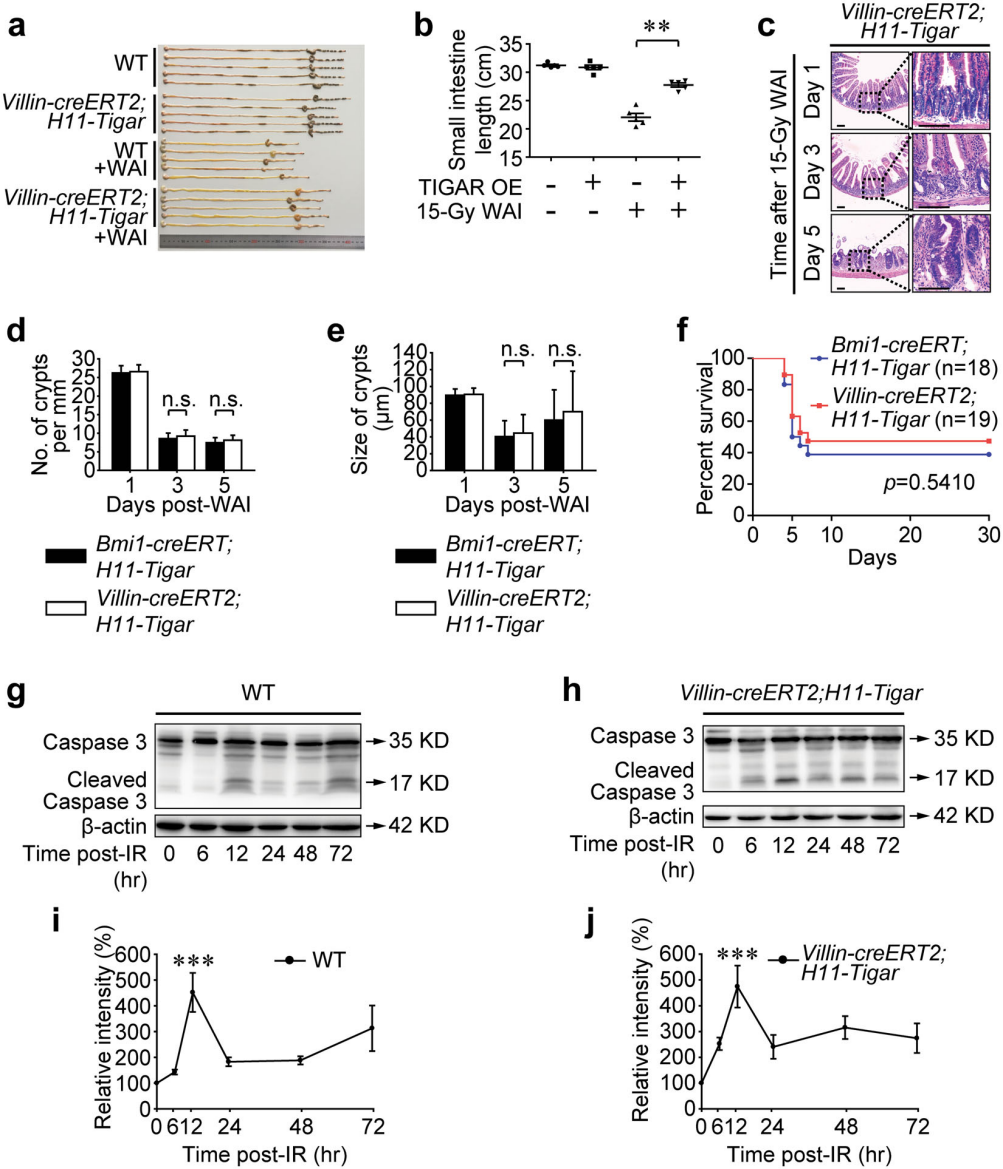
Intestinal TIGAR-overexpression displays equivalent reconstruction to restricted TIGAR-induction. **a-h** Gene targeting strategy for *Villin-creERT2;H11-Tigar* mice. **b** TIGAR is introduced by single intraperitoneal injection of tamoxifen immediately after 15-Gy WAI. **b** Image of small intestines 5 days post 15-Gy WAI. **c** The length of small intestines 5 days post-WAI. **p < 0.01. **c** Representative images of H&E staining of small intestines from *Villin-creERT2;H11-Tigar* mice at indicated time after 15-Gy WAI. Six sections per mouse, *n* = 3 animals. Scale bars = 100 μm. **d** Number of crypts per millimeter post-WAI. Values are expressed as mean ± SD. **e** Size of crypts after 15-Gy WAI. Values are expressed as mean ± SD. **f** Kaplan–Meier survival analysis of *Bmi1-creERT;H11-Tigar* and *Villin-creERT2;H11-Tigar* mice after 15-Gy WAI. **g**, **h** Western blot for Caspase-3 in isolated intestinal crypts from *H11-Tigar* mice and *Villin-creERT2;H11-Tigar* mice being irradiated by 15-Gy WAI. **i**, **j** Relat**i**ve intensity of Caspase-3 expression in isolated intestinal crypts from *H11-Tigar* mice and *Villin-creERT2;H11-Tigar* mice. Values are expressed as mean ± SD. ***p < 0.001 (12 h vs. 0 h).

To determine whether TIGAR-overexpression within the entire intestinal epithelium could display a better regenerative response than restricted induction in reserve ISCs, *Villin-creERT2;H11-Tigar* mice were exposed to 15-Gy WAI. Based on this animal model, TIGAR could be induced simultaneously in both *Bmi1-creERT*^+^ ISCs and *Lgr5*^+^ secretory progenitor cells, and the potential synergistic effects on crypt regeneration of these TIGAR-overexpressing cells could be examined. Exactly, *Villin-creERT2;H11-Tigar* mice revealed a notable attenuation in intestinal length shortening (Fig. 5a, b) and a considerable amelioration in epithelial integrity (Fig. 5c) after 15-Gy WAI. Critically, the crypt regeneration (Fig. 5d, e) and survival rate (Fig. 5f) of *Villin-creERT2;H11-Tigar* mice resembled those of *Bmi1-creERT;H11-Tigar* mice after lethal irradiation, indicating that TIGAR-induction failed to promote *Lgr5*^+^ secretory progenitor cell division to support crypt regeneration, even if the reserve ISCs were already accelerated to proliferation. Furthermore, the data also revealed that the contribution of other epithelial populations to crypt regeneration might be vanishingly small. Actually, although crypt cells such as *Alpi-CreER*-marked TA cells are reported to repopulate the crypt compartment upon IR-injury, little evidence supports their functional importance in epithelial regeneration^28^. Meanwhile, IR-evoked apoptosis of intestinal crypts within 24 h post-WAI was almost the same no matter TIGAR was overexpressed or not (Fig. 5g–j), suggesting that the post-IR treatment applied in the current study failed to attenuate WAI-induced crypt cell death. Hence, it was summarized that the amelioration of intestinal integrity induced by TIGAR-overexpression after lethal IR-injury was predominantly attributed to the acceleration of *Bmi1-creERT*^+^ reserve-ISC division.

## Discussion

A two-stem cell model is supported by burgeoning studies from the small intestine, involving an actively cycling but radiosensitive stem cell and a long-lived, injury-resistant reserve pool of ISCs which is regarded to reside upstream of the high-proliferating CBCs^11,24,29,30^. Classic theories of radiobiology demonstrate that cell’s radiosensitivity is positively correlated with the proliferative activity. Indeed, relieving the proliferative suppression of the reserve pool of ISCs before irradiation can result in enhanced epithelial radiosensitivity and aggravated GIS^16^. Conversely, if the suppressed cell division of low-proliferating stem-like cells cannot be released in time, the intestinal integrity may also fail to be regenerated after lethal IR-injury. Using “*cre-loxp*” mouse model, the present study indicates the possibility of TIGAR-based post-IR treatment in accelerating reserve-ISC division and ameliorating mouse survival under grievous GIS.

To establish the involvement of TIGAR in driving the intestinal regeneration, we applied mouse models of which TIGAR could be efficiently induced 18 h after stimulation in vivo (Supplementary Fig. 4a–f). During WAI, the head, neck, thorax, and extremities were shielded to protect the bone marrow (Fig. 1b), thus inducing predominant GIS^3,4^. Single intraperitoneal injection of tamoxifen was performed immediately after 15-Gy WAI to induce TIGAR expression timely. After lethal WAI exposure, *Bmi1-creERT;H11-Tigar* and *Villin-creERT2;H11-Tigar* mice revealed equivalent amelioration of intestinal epithelial integrity (Fig. 5c–e) and mouse survival (Fig. 5f), indicating that TIGAR-overexpression primarily accelerated reserve ISCs toward division to reestablish the intestinal architecture after lethal irradiation. It is worth noting that biomarkers of “quiescent” reserve ISCs are also found in a subpopulation of *Lgr5*^+^ crypt cells, while around 20% of *Lgr5*^+^ intestinal cells are largely quiescent^13,24^. This quiescent *Lgr5*^+^ population is mainly comprised of the *Lgr5*^+^ (label-retaining) secretory progenitors and a subpopulation of the *Bmi1-creERT*^+^ reserve ISCs. Critically, by lineage cell tracing analysis, the feasibility of TIGAR-overexpressing quiescent *Lgr5*^+^ cells in rescuing mice from lethal GIS was ruled out (Fig. 2i–k; and Supplementary Fig. 1f–h). The mechanisms might be roughly attributed to the following two reasons. On the one hand, when compared with the exact quiescent *Bmi1-creERT*^+^ reserve ISCs, quiescent *Lgr5*^+^ cells were reported to demonstrate much fewer tracing events in response to injury^13,14^, which made effective crypt regeneration incapable after lethal WAI exposure. On the other hand, the *Lgr5*^+^ characteristics endowed these cells with higher radiosensitivity^31^, which made them already lose viability or undergo apoptosis before TIGAR was introduced (Fig. 5h, j). However, the present study does not eliminate the indispensability of the de novo-generated *Lgr5*^high^ CBCs in intestinal regeneration after lethal IR-injury^32^.

**Fig. 6:**
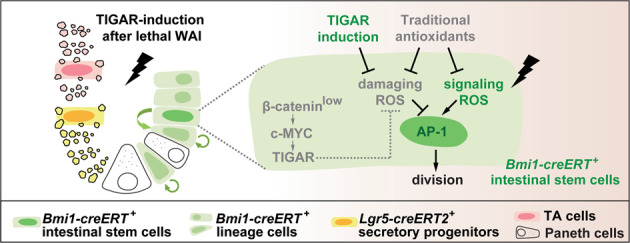
A proposed model illustrating cell division geared by TIGAR-induction after lethal WAI. After lethal irradiation, TIGAR-induction accelerates cell division of *Bmi1-CreERT*^+^ reserve ISCs and facilitates crypt regeneration (left). By eliminating damaging ROS yet retaining signaling ROS which is indispensable for proliferation, TIGAR-overexpression notably enhances the AP-1 activity and gears *Bmi1-CreERT*^+^ cell division after lethal IR-injury (right).

Based on the lineage tracing analysis, a recent study indicated that the *Bmi1*^+^ cancer stem cells possessed an increased AP-1 activity that drove tumor recurrence^33^, suggesting that AP-1 played critical roles in endowing *Bmi1-creERT*^+^ stem cells with proliferative potential. In the present study, a classical inhibitor of AP-1, 3-PA, was used to demonstrate the mechanism of TIGAR-induced proliferation after lethal IR. A significant abrogation of *Bmi1-creERT*^+^ cell division, especially the first asymmetric division at the early stage (1 day) post-IR, was observed when the transcriptional activity of AP-1 was inhibited by 3-PA (Fig. 3j, k). Interestingly, AP-1 abolishment only dramatically abrogated the *Bmi1-creERT*^+^ lineage after irradiation, but did not affect the proliferative activity of CBCs during homeostatic conditions (Supplementary Fig. 2c, d). This finding suggests that the AP-1 activity is dispensable for CBC-like stem cells during homeostasis, which might attribute to the high proliferative activities of *Lgr5*^high^ CBCs endowed by the Wnt/β-catenin signals^31,34–36^. This also suggested that TIGAR-induction remedied the β-catenin-inactivated “defect” of the low-proliferating reserve ISCs, which facilitated the acceleration of cell division and crypt regeneration after lethal IR-injury (Fig. 6). Mechanically, TIGAR-induced activation of c-Fos/AP-1 might be attributed to the increased phosphorylation of c-Fos, but not the upregulation of c-Fos expression.

In conclusion, the current study indicates that during unexpected disasters, quiescent *Lgr5*^−^ reserve ISCs can be awakened timely by TIGAR/AP-1 activation to reestablish intestinal architecture and ameliorate mouse survival. Meanwhile, our work reveals an unexplored role of TIGAR in accelerating reserve ISCs toward regeneration, and the capability of TIGAR-induction in activating AP-1 demonstrates its significant advantage over traditional antioxidant treatments (Fig. 6).

## Materials and methods

### Experimental model

Mature male and female *Villin-creERT2* mice (B6N.Cg-Tg(Vil1-cre/ERT2)23Syr/J), *Bmi1-creERT* mice (B6;129-*Bmi1*^*tm1(cre/ERT)Mrc*^/J), *Lgr5-EGFP-IRES-creERT2* mice (B6.129P2-*Lgr5*^*tm1(cre/ERT2)Cle*^/J), *Rosa26-EYFP* mice (B6.129×1-*Gt(ROSA)26Sor*^*tm1(EYFP)Cos*^/J) and *Rosa26-mTmG* mice (B6.129(Cg)-*Gt(ROSA)26Sor*^*tm4(ACTB-tdTomato,-EGFP)Luo*^/J) were purchased from Jackson Laboratory (ME, USA). *H11-Tigar* mice (C57BL/6N-*Igs2*^*em1(CAG-LSL-Tigar-2A-EGFP)Cgen*^), (i.e., mutant mice carry a *CAG-loxp-stop-loxp-Tigar-2A-EGFP-polyA* insertion targeted to the mouse *Igs2* (intergenic site 2; *H11*) locus) were generated by Cyagen Biosciences Company (Guangdong, China). All mouse lines were maintained and bred on mixed genetic background.

If not otherwise stated, only male mice were used and littermates were randomly and blindly allocated to experimental groups. All experiments were conducted at 8–10 weeks of age. Genotyping was performed following the protocols of Jackson Laboratory. The study was conducted in compliance with local animal welfare laws, guidelines, and policies. All procedures were approved by the ethic committee of Soochow University (Approval No. ECSU-2019000150).

### Mouse irradiation and tamoxifen administration

Mice weighing between 24 and 27 g were anesthetized and treated with a single dose of 15-Gy WAI at a dose rate of 1.6 Gy/min using an X-RAD 320iX Biological Irradiator (Precision X-ray, North Branford, CT, USA). A 3-cm area of the mice containing the gastrointestinal tract was irradiated (irradiation field), shielding the head, neck and upper thorax as well as lower and upper extremities and protecting a significant portion of the bone marrow. Immediately after irradiation, mice were injected with tamoxifen intraperitoneally. Tamoxifen (Sigma, Cat#T5648) was dissolved in corn oil (Sigma, Cat#C8267) at a final concentration of 20 mg/ml. Cre enzyme was induced by single injection of tamoxifen at a dose of 4.5 mg per 20 g body weight. The schedules for tamoxifen administration and radiation as well as mouse grouping were provided in the relevant figure legends. If not otherwise stated, *H11-Tigar* mice were used as controls and were given similar doses of tamoxifen.

### Survival rate and small-intestine length

After 15-Gy WAI, the survival rate of the mice was monitored every day for up to 30 days. For intestinal length measurement, mice died before day 5 post-WAI were excluded, and the mice alive were administered with euthanasia on day 5 after 15-Gy WAI to analyze the small intestinal length.

### Histology of small intestine

At day 1, 3, 5 after 15-Gy WAI, mice were administered with euthanasia, and the proximal small intestines were excised for histology. Small intestine tissues were fixed in 10% neutral-buffered formalin overnight. After embedded in paraffin, tissues were cut into 5-μm sections for haematoxylin and eosin (H&E) staining and observation.

### Western blot assay

Protein lysates were prepared in RIPA buffer (Thermo Fisher Scientific, Cat#89900), resolved via SDS-PAGE, and transferred onto polyvinylidene fluoride membranes (Millipore, Cat#ISEQ00010). The following primary antibodies were used: TIGAR (Abcam, Cat#ab62533), Caspase-3 (CST, Cat#9662), β-actin (Abcam, Cat#ab8226). After incubation with HRP-conjugated secondary antibodies, bands were detected and visualized by FluorChem M (ProteinSimple, San Jose, CA, USA).

### Crypt isolation

Mouse small intestine was cut open longitudinally and washed with cold PBS. The villi were collected by scraping the intestine with a microscope slide and stored for protein analysis. While the remaining small intestine was cut into 5-mm pieces and incubated in PBS with 2 mM EDTA for 30 min at 4 °C. After incubation, the tissue fragments were separated by vigorous shaking. The supernatant enriched for crypts was passed through a 70-μm cell strainer (BD Falcon, Cat#352350). After centrifugation, the final fraction of intestinal crypts was used for further culture.

### Organoid culture

Isolated crypts were suspended with Matrigel (Corning, Cat#356231, growth factor reduced), which was overlaid by the organoid medium. Advanced DMEM/F12 (Thermo Fisher Scientific, Cat#12634-010) medium was supplemented with EGF 40 ng/ml (Peprotech, Cat#315-09-100), Noggin 100 ng/ml (Peprotech, Cat#250-38-5), R-spondin 500 ng/ml (Peprotech, Cat#315-32-5), N2 1× (Thermo Fisher Scientific, Cat#17502048), B27 1× (Thermo Fisher Scientific, Cat#17504044), and Y-27632 dihydrochloride monohydrate 10 μM (Sigma, Cat#Y0503). Intestinal crypts were cultured in the above mentioned media in Matrigel until further assay. For TIGAR-induction, 4-OHT (Sigma, Cat#T176) with a concentration of 10 nM was administrated in vitro. After 24 h, the medium was replaced by normal organoid culture medium, with organoids still in Matrigel.

### Organoid irradiation and transfection

After 12-Gy irradiation in vitro, transfection of *Tigar*-overexpressing adenovirus (Adv-*CMV*-*Tigar*-*3flag*) was performed by mechanical separating the organoids from Matrigel, and 4-OHT (10 nM) was added into the medium soon after replanting the organoids. After 24 h, the medium was replaced by normal organoid culture medium.

### Immunofluorescence assay

After culturing, the intestinal organoids were planted on 35-mm glass bottom dishes (Thermo Fisher Scientific, Cat#150682) and fixed by 4% cold paraformaldehyde, washed with cold PBS and blocked in Blocking buffer (1× PBS, 5% anti-goat serum, 0.01% Triton X-100) for 1 h. Then the fixed organoids were successively incubated with anti-DDDDK tag primary antibodies (Abcam, Cat#ab1162) or anti-Ki67 primary antibodies (Abcam, Cat#ab15580) at 4 °C overnight and secondary antibodies (Abcam, Cat#ab150073) or (Abcam, Cat#ab150062) at room temperature for 90 min. For frozen-section staining, optimal cutting temperature compound (Leica, Cat#14020108926) embedded intestinal cryosections of 10–14 μm thickness were stained with the DAPI for 5 min at room temperature. The confocal images were acquired using an Olympus FV1200 confocal microscope (Japan).

### Fluorescence-activated cell sorting (FACS)

The intestine was cut open longitudinally and incubated with 2 mM EDTA solution at 4 °C for 30 min to isolate intestinal crypts. To generate a single cell suspension, cells were incubated with Accutase (BD Biosciences, Cat#561527) at 37 °C for 10 min. Flow cytometry analysis was performed with CELL SORTER SH800S (SONY, Japan). Cells were gated for single cell based on the profiles of Forward-scatter area versus backward-scatter area (FSC-A vs. BSC-A) and forward-scatter height versus forward-scatter width (FSC-H vs. FSC-W). The size of the nozzle for all sorting is 100 µm.

### Analysis of AP-1 activation

The DNA-binding activity of AP-1 was measured using the Trans^AM^ kits (Active Motif, Cat#44096). Nuclear extracts of *Bmi1-creERT*^+^ cells containing c-Fos/AP-1 factors were added into the multi-well plates precoated with consensus double-stranded DNA oligomers. After incubation, the transcription factor bound to DNA sequences was detected by using antibodies against c-Fos according to the manufacturer’s protocol. The absorbance was examined by a Microplate Reader (BioTek, Synergy2, Winooski, VT, USA).

### 3-PA and NAC treatments

3-PA (MedChem Express, Cat#HY-12270) was dissolved in polyvinylpyrrolidone. For in vivo administration, mice were administrated with 3-PA (120 mg/kg body weight, i.g.) daily for 4 consecutive days before 15-Gy WAI and the subsequent tamoxifen induction. For in vitro administration, 3-PA (10 μM) was added into the organoid growth medium 1 day before 12-Gy irradiation. For NAC treatment, the organoids were treated with NAC (Sigma, Cat#A8199) soon after the irradiation at concentrations of 1.5 mM or 4.0 mM, respectively.

### Statistical analysis

Data were expressed as mean ± SD from three independent determinations. Differences between groups with similar variance were analyzed by Student’s *t* test. Kaplan–Meier survival analysis and log-rank comparison were performed for survival studies. Asterisks represent the *p* values as follows: *p < 0.05, **p < 0.01 and ***p < 0.001.

## Supplementary information

Supplementary Figure Legends

Supplementary Figure S1

Supplementary Figure S2

Supplementary Figure S3

Supplementary Figure S4
