# Supplementary material for: TIGAR/AP-1 axis accelerates the division of Lgr5− reserve intestinal stem cells to reestablish intestinal architecture after lethal radiation
Source: Cell Death Dis. 2020 Jul 6;11(7):501. doi: 10.1038/s41419-020-2715-6 (PMC7338449; doi:10.1038/s41419-020-2715-6)
Supplement: Supplementary file 1 — Supplementary Figure Legends [file 41419_2020_2715_MOESM1_ESM.doc]

**Supplementary Figure Legends**

**Supplementary Fig. 1** TIGAR-induction in *Lgr5*^+^ secretory progenitor cells fails to regenerate crypts after lethal IR. **a** Gene targeting strategy for *Lgr5-creERT2;H11-Tigar* mice. **b**-**e** TIGAR is introduced by single intraperitoneal injection of tamoxifen immediately after 15-Gy WAI. **b** Representative images of H&E staining of small intestines from *Lgr5-creERT2;H11-Tigar* mice (right panel) and their WT cohorts (left panel) after 15-Gy WAI. Six sections per mouse, n = 3 animals. Scale bars = 100 μm. **c** Number of crypts per millimeter (left) and the size of crypts (right) after 15-Gy WAI. Values are expressed as mean ± SD. **d** Image of small intestines from *Lgr5-creERT2;H11-Tigar* mice and their WT cohorts 5 days post-IR. **e** Length of small intestines 5 days post-WAI. **f** Lineage tracing analysis of cultured intestinal organoids derived from *Lgr5-creERT2;H11-Tigar* mice. A concentration of 10 nM of 4-OHT is added into the culture medium immediately after 12-Gy irradiation. Fluorescence microscopy reveals the *Lgr5-creERT2^+^* cells at indicated time after IR. White dashed lines indicate a single cell. Scale bars = 50 μm. **g** Percentage of GFP positive organoids from *Lgr5-creERT2;H11-Tigar* mice. Values are expressed as mean ± SD. **h** Number of GFP positive cells per organoid at indicated time post-IR. Values are expressed as mean ± SD. * *p* < 0.05, *** *p* < 0.001.

**Supplementary Fig. 2** 3-PA fails to attenuate the proliferation of CBCs during homeostatic conditions. **a**, **b** Schematic diagrams show the experimental strategies for co-treatments of irradiation and 3-PA *in vivo* and *in vitro*. **c** Intestinal organoids are derived from isolated crypts from *Lgr5-creERT2;H11-Tigar* mice and are cultured *in vitro*. Organoids are pretreated with 3-PA (10 μM) one day before TIGAR-introducing. Fluorescence microscopy illustrates the fluorescence-positive organoids at indicated time after 4-OHT induction. Scale bars = 50 µm. **d** Number of fluorescent cells per organoid from *Lgr5-creERT2;H11-Tigar* mice at indicated time after TIGAR-induction. Values are expressed as mean ± SD. *** *p* < 0.001.

**Supplementary Fig. 3** Redundant TIGAR activity fails to gear reserve ISCs toward division during homoeostasis. **a**, **b** Intestinal organoids are derived from isolated crypts from *Bmi1-creERT;H11-Tigar* mice and their WT cohorts and cultured for 2 days *in vitro*. On Day 3, TIGAR is induced by 4-OHT (10 nM). Organoids are cultured for another 5 days after TIGAR-induction *in vitro*. **a** The morphology of WT and TIGAR-overexpressing organoids is illustrated. Scale bars = 300 μm. **b** Quantiﬁcation of the number of buds from WT and TIGAR-overexpressing (OE) crypt cultures.

**Supplementary Fig. 4** TIGAR is efficiently induced 18 hr after stimulation *in vivo*. **a** Representative frozen-sections of proximal intestine derived from *Villin-creERT2;H11-Tigar* mice. TIGAR is introduced by single intraperitoneal injection of tamoxifen for indicated time. Six sections per mouse, n = 3 animals. Scale bars = 100 μm. **b** Percentage of GFP positive crypts at indicated time after tamoxifen-induction. Values are expressed as mean ± SD. **c**-**f** TIGAR expression in *Villin-creERT2;H11-Tigar* mice and their WT cohorts. TIGAR is introduced by single intraperitoneal injection of tamoxifen for 18 hr. **c** Western blots for TIGAR expression in small intestine and colon tissues. **d** Relative intensity of TIGAR expression level in small intestine and colon tissues. Values are expressed as mean ± SD. **e** Western blots for TIGAR expression in villi and crypts of small intestines. **f** Relative intensity of TIGAR expression level in villi and crypts of small intestines. Values are expressed as mean ± SD. *** *p* < 0.001.
