# Supplementary figures and images for: TIGAR/AP-1 axis accelerates the division of Lgr5− reserve intestinal stem cells to reestablish intestinal architecture after lethal radiation
Source: Cell Death Dis. 2020 Jul 6;11(7):501. doi: 10.1038/s41419-020-2715-6 (PMC7338449; doi:10.1038/s41419-020-2715-6)

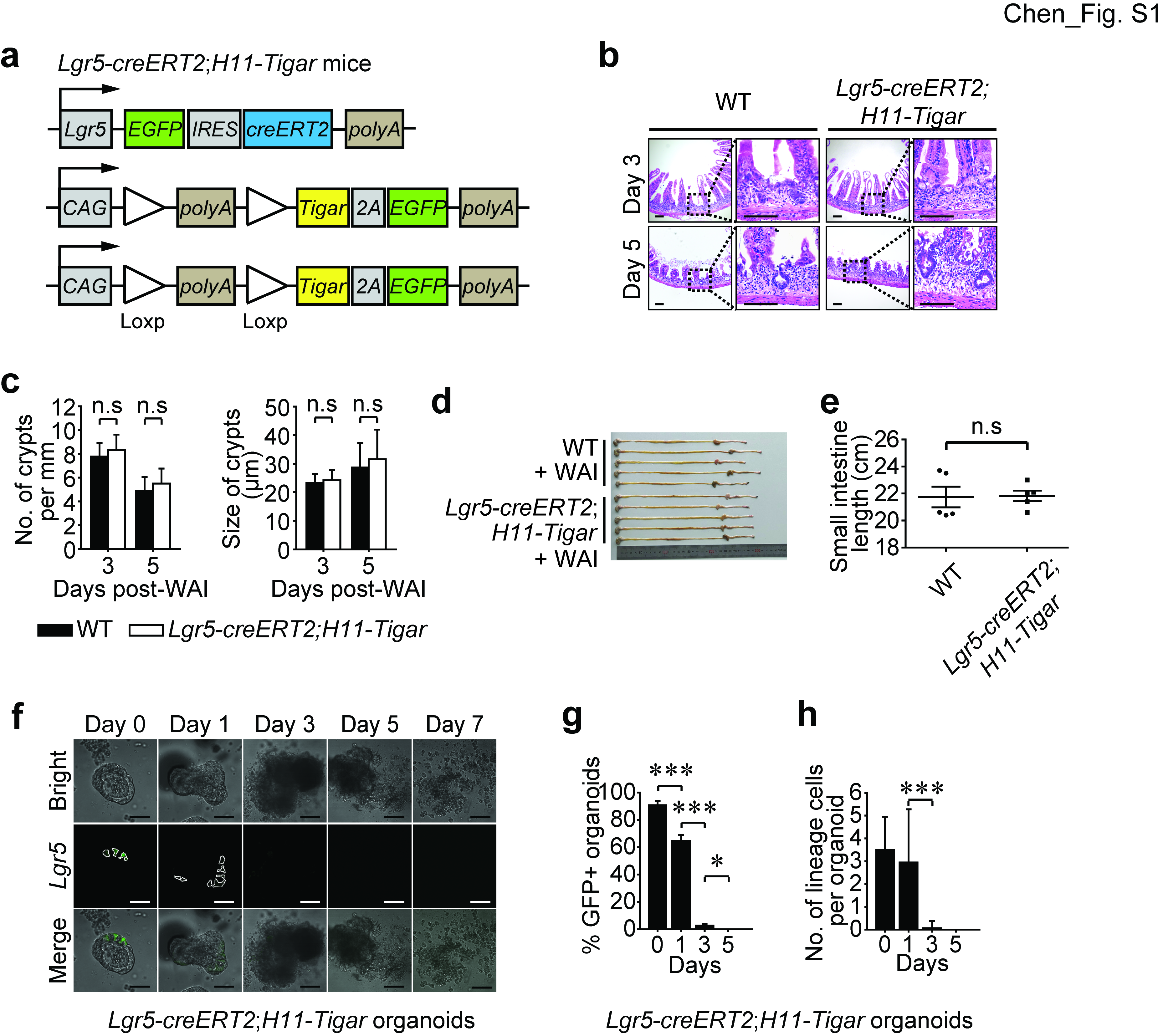

Supplement: Supplementary file 2 — Supplementary Figure S1 [file 41419_2020_2715_MOESM2_ESM.tif]

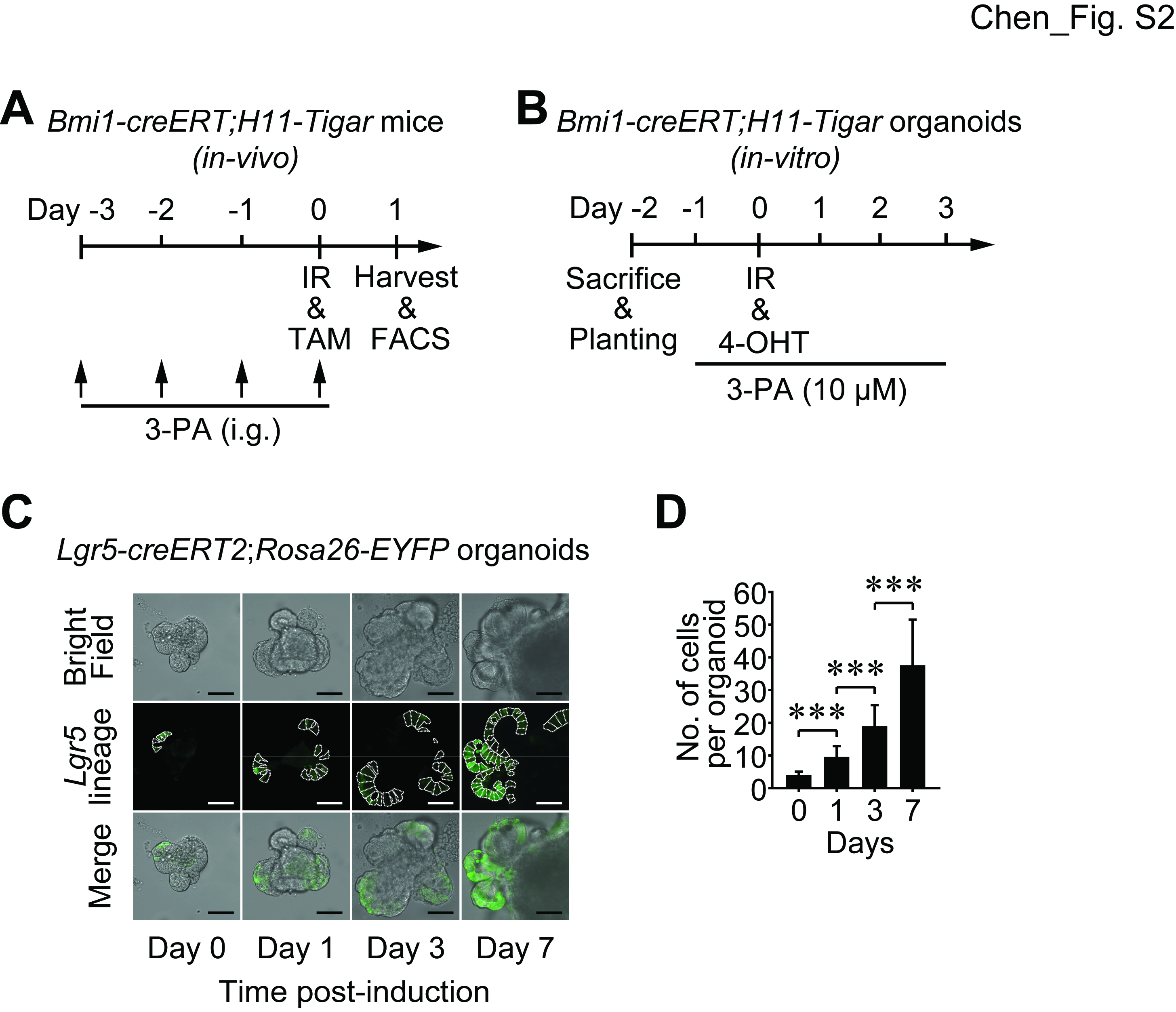

Supplement: Supplementary file 3 — Supplementary Figure S2 [file 41419_2020_2715_MOESM3_ESM.tif]

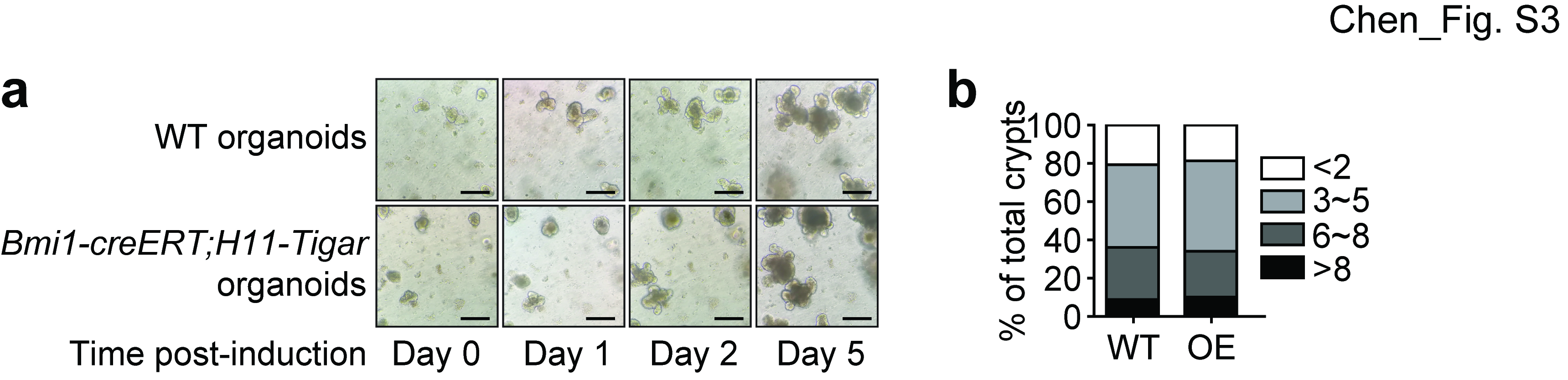

Supplement: Supplementary file 4 — Supplementary Figure S3 [file 41419_2020_2715_MOESM4_ESM.tif]

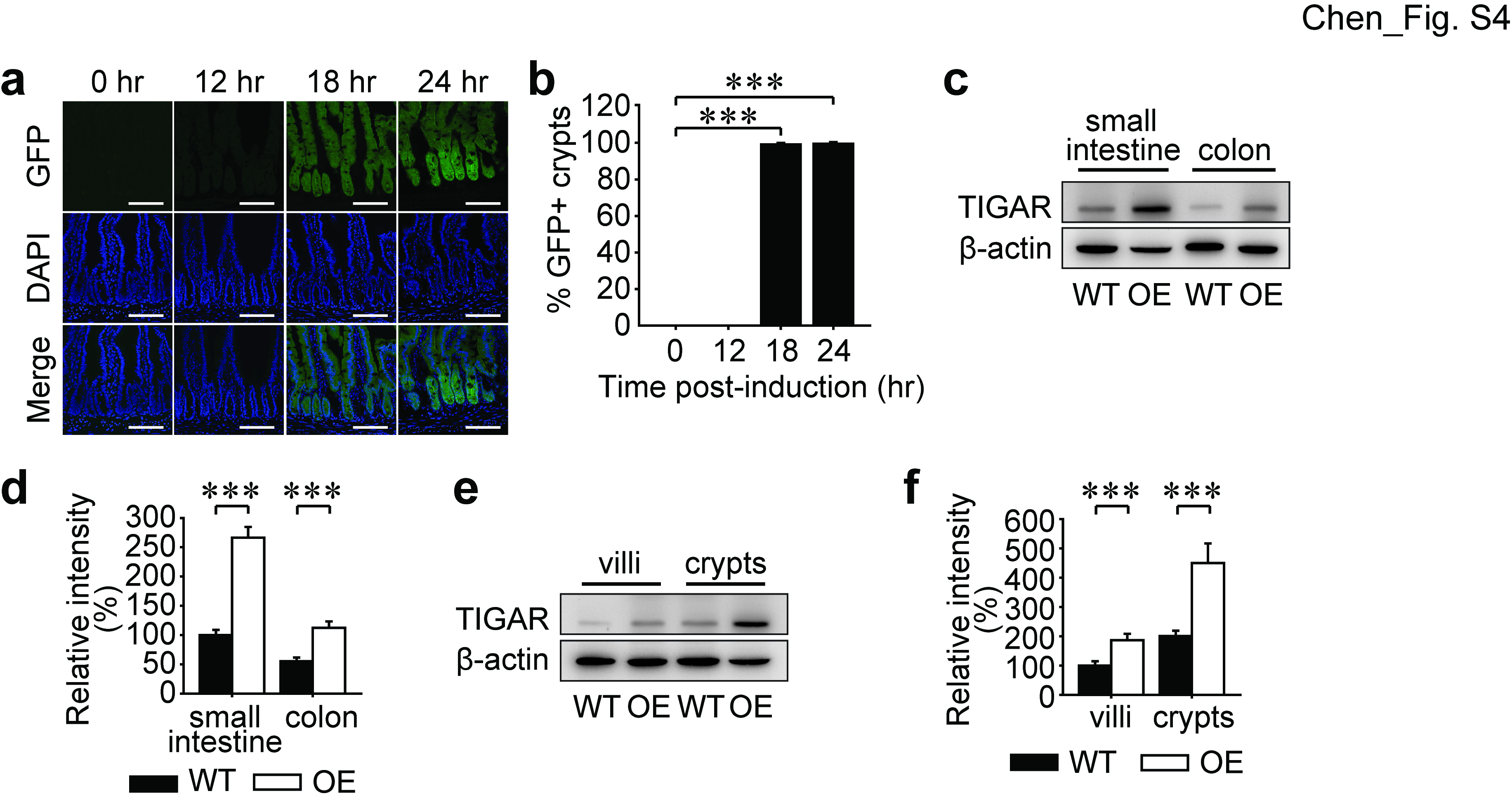

Supplement: Supplementary file 5 — Supplementary Figure S4 [file 41419_2020_2715_MOESM5_ESM.tif]
